# 

**Published:** 1883-08

**Authors:** 


ESTABLISHED IN 1855.

OLD SERIES, Vol. XXI—No. 5. AUGUST, 1883. NEW SERIES Vol. II—No. 11

### ATLANTA

### MEDICAL REGISTER.

*(ATLANTA MEDICAL AND SURGICAL JOURNAL.)*

EDITED BY

J. H. LOGAN, A.M., M.D.

H. H. DICKSON, PUBLISHER. TERMS, $2.50 A YEAR IN ADVANCE.

ATLANTA, GEORGIA:

*H. H. DICKSON, BOOK AND JOB PRINTER AND BOOKBINDER.*

1883.

